# Detection of the Vascular Endothelial Growth Factor with a Novel Bioluminescence Resonance Energy Transfer Pair Using a Two-Component System

**DOI:** 10.3390/s17010145

**Published:** 2017-01-13

**Authors:** Tobias Wimmer, Eva Schroeter, Birgit Lorenz, Knut Stieger

**Affiliations:** Department of Ophthalmology, Justus-Liebig-University Giessen, Friedrichstr. 18, 35390 Giessen, Germany; tobias.wimmer@augen.med.uni-giessen.de (T.W.); eva_schroeter@hotmail.de (E.S.); Birgit.Lorenz@uniklinikum-giessen.de (B.L.)

**Keywords:** VEGF, neuropilin, BRET, ranibizumab, single chain variable fragment (scFv), RLuc8, PerCP-Cy5.5^®^

## Abstract

In this paper we describe a two-component BRET (bioluminescence resonance energy transfer)-based method to detect vascular endothelial growth factor (VEGF) molecules in unknown samples as the basis for subsequent in vivo use. A luminescent VEGF binding molecule, which binds in the receptor binding motif of VEGF, is used as the energy donor, transferred to a fluorophore-coupled VEGF binding molecule (acceptor), which binds to the neuropilin binding motif of VEGF, thus enabling energy transfer from the donor to the acceptor molecule. This leads to the emission of light at a longer wavelength and thus the generation of an increased BRET signal only when VEGF is bound to both the donor and acceptor molecules. We further describe a novel BRET pair that uses the *Renilla reniformis* mutant luciferase RLuc8 and the chemically engineered fluorophore PerCP-Cy5.5^®^, which exhibits superior peak separation of approximately 300 nm. The implantation of capsules consisting of the two BRET components in solution, permeable for VEGF for its in vivo detection, would provide a new and improved method for monitoring VEGF-induced pathologies and thus an adjustment of therapy to patient needs.

## 1. Introduction

Resonance energy transfer (RET), a principle first characterized by Theodore Foerster in 1948, describes the transfer of energy by spectral overlapping [1]. Fluorescence resonance energy transfer (FRET) has expanded applications in laboratories through the development of coupling fluorescent probes to biomolecules such as proteins or DNA [2]. FRET uses a fluorescent donor which transfers energy to a suitable fluorescent acceptor by overlapping the donor’s emission wavelength with the acceptor’s excitation wavelength, which emits light again at higher wavelengths. Bioluminescence resonance energy transfer (BRET) uses the same principle, but the donor is replaced by a bioluminescent luciferase instead of a fluorescent donor. The bioluminescent luciferase, as a catalytic enzyme, produces a light emission by converting its substrate, and generates an emission peak that can be used to excite a fluorophore [3]. Several suitable BRET pairings have been described with different donor luciferases (e.g., *Renilla reniformis* luciferase, firefly luciferase or click beetle luciferase) using different substrate analogues for light/energy generation and different acceptors (e.g., protein fluorophores or chemical fluorophores) (Table 1) [3,4,5,6]. The efficacy of energy transfer depends on how close the maximum of the donor emission overlaps the maximum of the acceptor excitation, the proximity of the donor to the acceptor and the distance between the donor and the acceptor. [7]. The measured acceptor signal, generated by energy transfer, depends on the spectral separation of the maximum donor and acceptor emission peaks (∆λem) and the orientation of the donor to the acceptor [4,5,8]. BRET systems have been widely used to understand and monitor protein-protein interactions such as in G-protein–coupled tyrosine kinase receptors, conformational changes in proteins, the quantification of protein binding molecules in vitro and in vivo or analyzing protease activity [2,7,8,9,10,11]. 

Personalized medicine is the current key word in optimizing therapy to patient needs in terms of diagnosis-correlated therapy. Therefore, “labs on a chip” or biosensors are currently under development to achieve improved, personalized therapy options for a variety of different disorders.

Especially in neovascular diseases of the eye such as age-related macular degeneration (AMD), retinopathy of prematurity (ROP), and diabetic retinopathy (DR), treatment personalization would be of great benefit to the patients. A typical feature common to these diseases is the hypoxia-induced upregulation of vascular endothelial growth factor (VEGF) expression which leads to the formation of new, but immature blood vessels in the eye which may impair visual function. VEGF, as a strong mitogenpromoting vessel growth, consists of three different receptor binding motifs enabling VEGF binding to VEGF receptor −1, −2 and neuropilin, which has been supposed to act as a co-receptor stabilizing VEGF receptor binding due to a lack of intracellular signaling [12]. Newer findings describe the neuropilin receptor as an activator of the ABL1 pathway [13]. Neuropilin binding is common to all angiogenic VEGF-A isoforms and is mediated through a C-terminal peptide sequence.

The current state-of-the-art treatment paradigm is a monthly injection of so-called anti-VEGF molecules (Avastin^®^, Lucentis^®^, EYELEA^®^), preventing VEGF binding to the VEGF receptors, thus decreasing activation of VEGF-induced intracellular signaling. The VEGF level varies from patient to patient and also during the disease course. The diagnostic parameters, such as visual acuity (VA) and ocular spectral optical coherence tomography (spectral OCT), used for monitoring the success of the therapy may indicate a rise in VEGF with delay, when VEGF inhibition is already decreasing. Another aspect is the different response to these anti-VEGF molecules which also varies among patients [14]. Many different methods for the detection and quantification of VEGF, such as ELISAs (enzyme linked immunosorbent assays) or biosensors also using different methods, have been described so far. All of them are designed for in vitro use after invasive sampling [15,16]. In this regard, a noninvasive, in vivo method to measure VEGF concentrations would greatly improve personalized anti-VEGF treatment regimes. 

**Figure 1:**
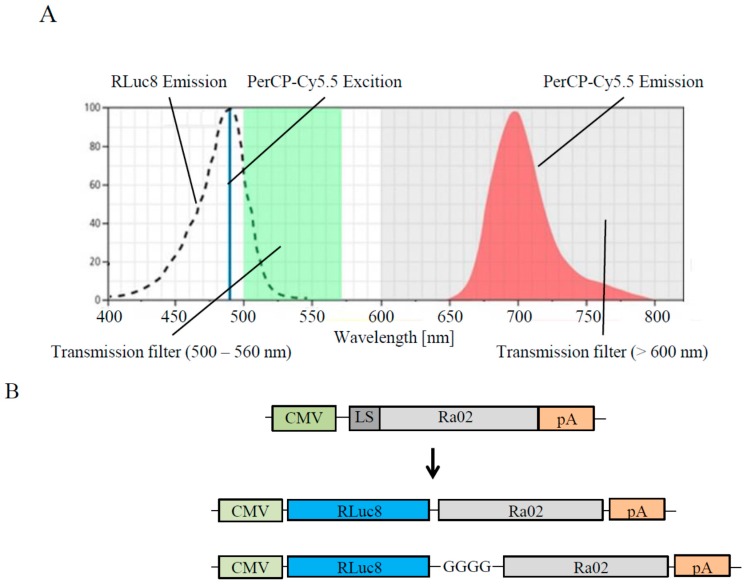
Schematic representation of the BRET principle and expression constructs. (**A**) RLuc8 converting n-CLZ and thus generating an emission peak (~490 nm) overlapping the excitation peak of PerCp-Cy5.5^®^, transferring energy to the fluorophore which is emitting light at ~700 nm; (**B**) BRET donor expression construct containing the luciferase RLuc8 and the VEGF binding domain Ra02 fused directly or separated with a 4× glycine peptide-linker, under the control of a CMV (cytomegalovirus) promoter and a polyadenylation signal (pA) sequence at the 3´-end.

The aim of this study was to establish a new BRET pair, consisting of a mutant of the *Renilla reniformis* luciferase (RLuc8) (λem = ~480 nm) in combination with its substrate coelenterazine (native or n-CLZ) as an energy donor with PerCP-Cy5.5^®^ (λex = ~488 nm, λem = ~700 nm), a chemically engineered fluorescent dye, as an energy acceptor (Figure 1A). RLuc8, a mutated wild type RLuc (A55T, C124A, S130A, K136R, A143M, M185V, M253L, S287L), with increased stability and quantum yield was used. RLuc8, the parental construct for random mutagenesis, resulted in the red-shifted variant RLuc8.6 [4]. RLuc8 was fused to a single-chain variable fragment (scFv) based on Ranibizumab (Ra02, [10]) which binds to the VEGF receptor binding motif of VEGF located at the *N*-terminus of VEGF and PerCP-Cy5.5^®^ coupled to the recombinant neuropilin receptor, which in turn binds to the C-terminal neuropilin motif of VEGF. The combination of this donor and acceptor exhibited superior spectral separation properties (∆λ = ~300nm; Table 1). Further, we tested different experimental setups applicable for VEGF detection based on the principle of the ALPHALISA^®^ technology (Perkin Elmer, Hamburg, Germany) where a dual antibody system is used for VEGF quantification. The system we propose for VEGF detection is meant to be applicable in vivo, immobilized in an implantable device in the eye to monitor actual VEGF levels in patients in order to adjust anti-VEGF doses to patient needs, i.e., to personalize the treatment of neovascular diseases.

## 2. Material and Methods

### 2.1. Preparation of BRET Constructs

An anti-VEGF single chain variable fragment (scFv) (Ra02) (Figure 1B) was used to generate the BRET donors by deletion of the secretory IgG kappa leader sequence from the anti-VEGF open reading frame (ORF), and by fusing RLuc8 either directly to the *N*-terminus or separated with a peptide linker (4× glycine).

The fluorescent dye PerCP-Cy5.5^®^ (Lightning Link, Cambridge, UK) was coupled to carrier free recombinant neuropilin-1 (NRP-1) (R&D Systems, Wiesbaden, Germany) according to manufacturer instructions to generate the BRET acceptor. In brief, 1 µL of LL-modifier was added to 10 µL of carrier-free neuropilin 1 (1.0 µg/µL). The whole sample was mixed and pipetted to the lyophilized Lightning Link mix. After incubation at room temperature for 3 h, 1 µL of LL-quencher FD reagent was added per 10 µL. After additional incubation for 30 min, the conjugates were ready for use or stored at −20 °C.

### 2.2. Cell Culture and BRET Donor Expression

HEK293 (ATCC: CRL-1573) were cultured in DMEM (Dulbeccos modified eagle media) supplemented with penicillin/streptomycin, l-glutamine (200 mM) and 10% fetal bovine serum (FBS) (all purchased from PAN Biotech, Aidenbach, Germany). The cells were transfected with the BRET donor plasmid DNA (4 µg each) using Lipofectamine 3000 (Life Technologies, Darmstadt, Germany). Expression was carried out at 37 °C, 5% CO_2_ in a humidified incubator for 24 h. After expression cells were washed twice with phosphate buffered saline (PBS) and collected in luciferase lysis buffer (Promega, Mannheim, Germany). After two additional freeze-thaw cycles in liquid nitrogen, the crude lysate was cleared with centrifugation at 4 °C at 14,000 *g* for 5 min. The supernatant was transferred into a fresh tube and stored at −20 °C prior further analysis.

### 2.3. RLuc8 Activity

Equal amounts (10 µL) of each BRET donor (RLuc8-Ra02 and RLuc8-4xGly-Ra02) containing lysate were analyzed for luciferase expression. Native coelenterazine (n-CLZ, NanoLight Inc., Pinetop, AZ, USA) stock (1.0 mg/mL in 100% ethanol) was diluted in BRET assay buffer (PBS supplemented with 1.0 g/L d-glucose-monohydrate (Roth, Karlsruhe, Germany), 0.1 g/L calciumchloride-dihydrate (Merck, Darmstadt, Germany) and 0.1 g/L magnesium chloride heptahydrate (Merck, Darmstadt, Germany)) and allowed to stabilize for 20 min. The lysates were plated onto COSTAR Lumiplates Flat White (Corning, Berlin, Germany) and 100 µL of the substrate containing BRET assay buffer were added to each BRET donor. Luciferase light output was measured immediately after substrate addition using Tecan Infinite M1000Pro (Tecan, Groeding, Austria) with an integration time of 1 s. 

### 2.4. Western Blot Analysis

Equal amounts of lysates (20 µL) were separated on a 10% Tris-HCL SDS-PAGE and transferred to a nitrocellulose membrane. Membrane was incubated with rabbit anti-RLuc (Biomol, Hamburg, Germany) as first antibody and goat anti-rabbit-IgG-HRP (Sigma Aldrich, Taufkirchen, Germany) as secondary antibody. Rabbit anti-GAPDH (glyceraldehyde-3-phosphate dehydrogenase) (R&D Systems, Wiesbaden, Germany) in combination with goat anti-rabbit-IgG-HRP was used as loading control. 

### 2.5. Experimental Setup VEGF Detection

**Scheme 1:**
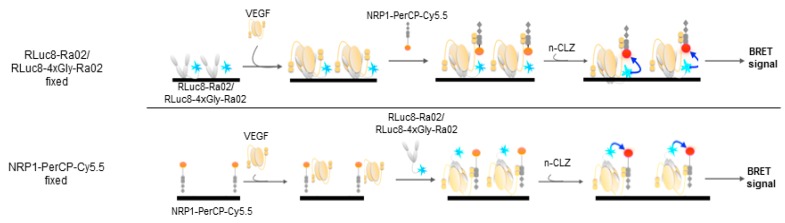
Donor and acceptor containing VEGF binding molecules are fixed onto a solid surface, following VEGF incubation and addition of the corresponding donor/acceptor molecule and possible generation of a BRET signal after addition of native coelenterazine (n-CLZ).

**Scheme 2:**
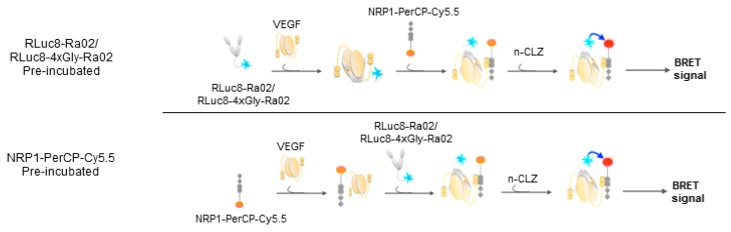
Donor and acceptor containing VEGF binding molecules are pre-incubated with VEGF, following the addition of the corresponding donor/acceptor molecule and possible generation of a BRET signal after addition of n-CLZ.

**Scheme 3:**
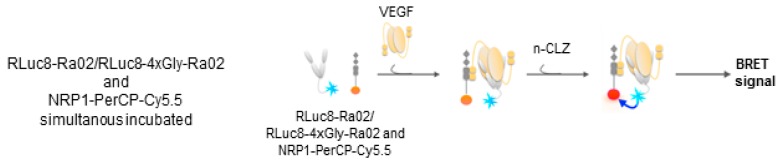
Donor and corresponding acceptor VEGF binding molecules are incubated simultaneously with VEGF and are generating a possible BRET signal after addition of n-CLZ.

According to the schematic representation (Scheme 1, Scheme 2 and Scheme 3 different setups for VEGF (rhVEGF165 produced in HEK293; Acro Bioscience; London, UK) detection were tested. 

Scheme 1, where the donor molecule or the acceptor molecule are fixed onto a solid surface (Nunc Maxisorb 96-well plates, Roskilde, Denmark), before a blocking procedure with 1% gelatin in 1× tris-buffered saline (TBS) and followed by incubation with VEGF and detection with the BRET donor/acceptor, according to the set-up of an ELISA (enzyme linked immunosorbent assay). Washing steps were performed with phosphate buffered saline (PBS) supplemented with 0.05% Tween 20 (Sigma Aldrich, Taufkirchen, Germany). 

Additionally to Scheme 1, a pre-incubated version where one of the two components (donor or acceptor), is incubated before the addition of VEGF and the second component (Scheme 2). In Scheme 3 both components were incubated simultaneously with VEGF. Both, donor and acceptor molecules were used in excess with a VEGF concentration of 22.4 nM (1.0 µg/mL). For the VEGF concentration depended measurements, VEGF was diluted with PBS, no VEGF control experiments were performed with PBS alone.

For every method, after the BRET pairs were incubated with VEGF 100 µL of the n-CLZ containing BRET assay puffer was added and readings were taken with the microplate reader (Tecan, Groeding, Austria) separately in the dual luminescence mode with both filters. BRET ratios were calculated according to Equation (1) and the no VEGF control was used to normalize the results. 

### 2.6. Measurement and Calculation of BRET Ratios

BRET measurements, after incubation with VEGF or no VEGF controls, were performed with two different transmission filters, a blue filter for the measurement of RLuc8 emission (500–560 nm) and a magenta filter for the detection of the PerCP-Cy5.5^®^ signal (>600 nm). BRET ratio calculations were done according the BRET Equation (1). Controls with no VEGF incubations were used for normalization of the BRET ratios. Mean BRET ratios of samples containing VEGF, or no VEGF as control, were calculated with measurements in triplicates plus standard deviation.

(1) $$\text{BRET Ratio}=\frac{\text{Biosensor}\;\left(\text{Magenta filter}\right)}{\text{Biosensor}\;\left(\text{Blue filter}\right)}$$

### 2.7. Statistics

All data are reported as mean ± standard deviation (SD). Experiments were measured in triplicates of at least two unrelated biological samples unless otherwise stated. Student´s *t*-test was used in all comparisons with SigmaPlot (Systat Software Inc.; Erkrath, Germany). *p*-values < 0.05 were considered as statistically significant.

## 3. Results and Discussion

### 3.1. Construct Generation, Expression and Functionality of Donor Molecules

The scFv Ra02 was used as the parental expression construct to generate the luminescent VEGF binding donor molecules [10]. The secretory IgG kappa leader sequence was deleted from the Ra02 VEGF binding domain open reading frame (orf) by PCR mutagenesis. The RLuc8 open reading frame was inserted at the 5′-end of the Ra02 orf, after the intrinsic stop codon was deleted, to generate the directly fused variant of the donor expression construct. The 4× glycine donor expression construct was generated with PCR mutagenesis by inserting a DNA sequence coding for 4× glycine (GGC GGA GGC GGA) (Figure 1B). Both expression construct orfs were confirmed by using Sanger sequencing.

**Figure 2:**
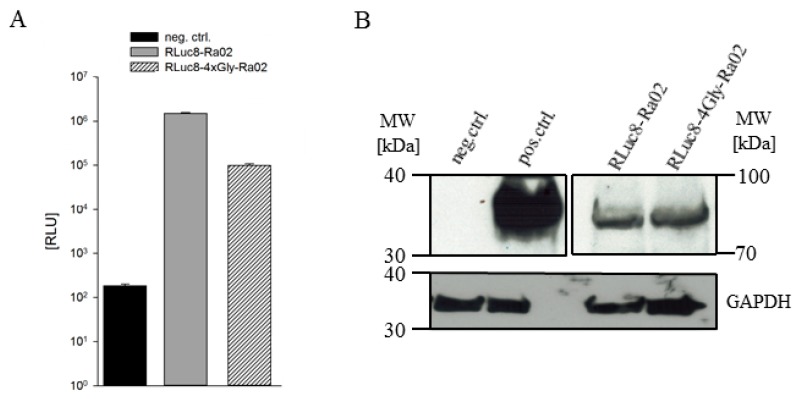
(**A**) RLuc8 activity of the donor molecule constructs expressed in HEK293 compared to a non-DNA transfection served as negative control (neg.ctrl.). RLuc8 transfection served as positive control (pos. ctrl.). Luciferase activities were measured in triplicate, two samples were used to calculate mean RLU ± SD. (**B**) Confirmation of full-length expression of the RLuc8 containing VEGF binding donor molecules. Expression compared to GAPDH.

After transfection and expression in HEK293 for 24 h, the activity of the RLuc8 was confirmed in a luciferase assay in crude but cleared cell lysate after the addition of 6.3 µM n-CLZ (Figure 2A). Both donor molecules as well as the RLuc8 alone, as a positive control, showed RLuc8 activity. Measured light outputs generated by the luciferases were 1494.933 × 10^3^ ± 74.120 × 10^3^ RLU for the directly fused RLuc8-Ra02 and 98.700 × 10^3^ ± 8.614 × 10^3^ RLU for the 4× glycine variant RLuc8-4xGly-Ra02. Negative control was measured as 185 ± 15 RLU. Due to the smaller size of the positive control, RLuc8 was expressed in higher amounts which led to a higher luciferase signal in this experiment as well as in the Western blot experiment, where equal amounts of cell lysates were separated.

Western blot analysis was performed to show the full-length expression of both BRET donor constructs. GAPDH (glycerinealdehyd-3-phosphat-dehydrogenase) was used as a loading control (Figure 2B). Expressed donor molecules were located, at the correct size of approximately 90 kDa, on the blot (calculated size: 87.9 kDa) and were expressed in nearly equal amounts compared to GAPDH.

These results confirm full-length expression as well as the ability of generating light/energy by coelenterazine conversion.

### 3.2. BRET Ratios of Different Experimental Setups

BRET ratios of the VEGF binding donor and the corresponding acceptor molecules were assessed for the three different experimental setups with VEGF or no VEGF as controls. 

**Figure 3:**
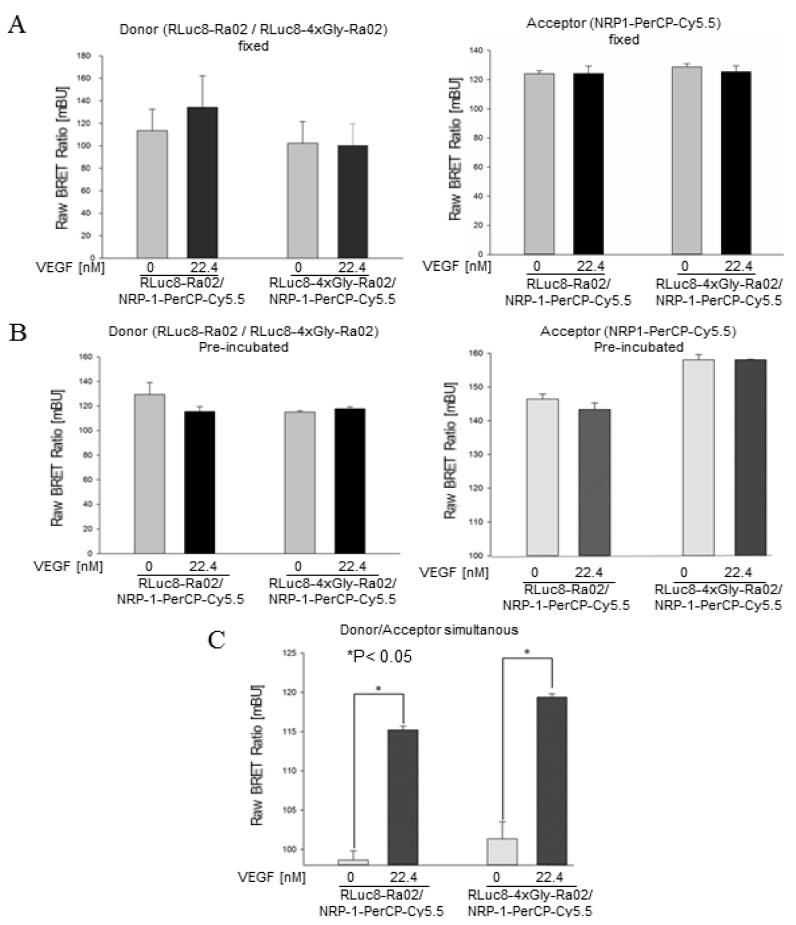
BRET ratios of the three different methods used after VEGF (22.4 nM) incubation and for corresponding no-VEGF controls. (**A**) Donor or acceptor molecules fixed onto a solid surface according to Scheme 1; (**B**) Donor or acceptor pre-incubation with VEGF according to Scheme 2; (**C**) Simultaneous donor and acceptor incubation with VEGF generating significantly different (*p* < 0.05) BRET ratios compared to no-VEGF controls for both donor molecule combinations. Plotted results were measured in triplicate, two unrelated biological samples were used to calculate mean values ± SD for Scheme 1 and Scheme 2.

Fixing the VEGF binding donor molecule or the VEGF binding acceptor molecule did not result in a significant increase of the BRET ratio after incubation with VEGF compared to the no-VEGF control for both donor molecules used (Figure 3A). The measured BRET ratios for the donor fixed setup were 1135 ± 189.5 mBU (milli BRET Units) in the no-VEGF control and 1341 ± 280.9 mBU after VEGF incubation for the combination of RLuc8-Ra02 and NRP1-PerCP-Cy5.5^®^, and 1023 ± 191.6 mBU for the control and 1004 ± 192.6 mBU after VEGF incubation for the RLuc8-4xGly-Ra02/NRP1-PerCP-Cy5.5^®^ combination. Fixing the acceptor molecule NRP1-PerCP-Cy5.5^®^ resulted in a BRET ratio of 124.1 ± 2.0 mBU for the no-VEGF control, and 124.2 ± 5.2 mBU after VEGF incubation for the RLuc8-Ra02 donor molecule. For the RLuc8-4xGly-Ra02 donor molecule, the BRET ratios were 128.7 ± 2.2 mBU for the control and 125.4 ± 4.1 for the VEGF sample.

Due to the unreproducible coating procedure of the molecules to the solid surface in combination with the following blocking procedure and the additional washing steps in this method, this setup is totally unsuitable for the reliable detection of VEGF in samples with unknown VEGF concentrations. Another explanation is that the VEGF molecules, as anti-parallel homo-dimers, have two binding motifs for Ra02 as well as for NRP1 [17]. In the first setup, there is the possibility that both Ra02 and NRP1 binding motifs were already saturated with just donor or acceptor molecules, making it impossible for the corresponding donor/acceptor molecule to bind to VEGF due to steric inhibition. VEGF was not able to bind NRP-1 after incubation/saturation with ranibizumab in a NRP-1 binding assay (unpublished data).

When VEGF was pre-incubated with the donor or the acceptor molecules, the BRET ratio also did not show a significant increase compared to the control for both donor molecules (Figure 3B). BRET ratios were calculated as 115.5 ± 3.9 mBU for the donor pre-incubated RLuc8-Ra02/NRP1-PerCP-Cy5.5^®^ combination incubated with VEGF and 129.4 ± 9.7 mBU for the no-VEGF control. The donor pre-incubated combination of RLuc8-4xGly-Ra02/NRP1-PerCP-Cy5.5^®^ was measured as 117.7 ± 1.6 mBU when incubated with VEGF and as 115.0 ± 1.1 mBU with no VEGF. 

Pre-incubation with the acceptor molecules resulted in similar results, 143.4 ± 1.9 mBU for the molecules pre-incubated with VEGF and 146.4 ± 1.5 mBU for the control using the RLUC8-Ra02 variant. 158.1 ± 0.1 mBU was measured for molecules using the RLUC8-4xGly-Ra02 variant for VEGF detection and 158.1 ± 1.5 mBU for the control. Here the effect of steric inhibition was more present than in the fixed method [16]. Although binding of these molecules is always described by a combination of association and dissociation, there is just little chance to replace one of the molecules with one another. Nevertheless, no measureable increase in the BRET ratio occurred due to this process for both donor molecules tested.

The simultaneous incubation of VEGF with both components of the biosensor system resulted in a significant increase (*p* < 0.05) in the BRET ratio due to the binding of the molecules and the transfer of energy from the donor molecule to the acceptor compared to the controls. In the case of the simultaneous incubation of VEGF with the VEGF binding donor and acceptor molecules, there is a higher chance for the two different molecules to bind to the same VEGF molecule and also a higher chance for detecting a BRET signal. Comparable affinity constants for donor and acceptor molecules are a requirement for consistent results in VEGF detection. The theoretical affinity constant for NRP1 to VEGF was determined as 0.3 pM and Ra02 was characterized with an affinity constant of 1.3 nM in our previous works on the scFv Ra02 [10,18,19].

**Figure 4:**
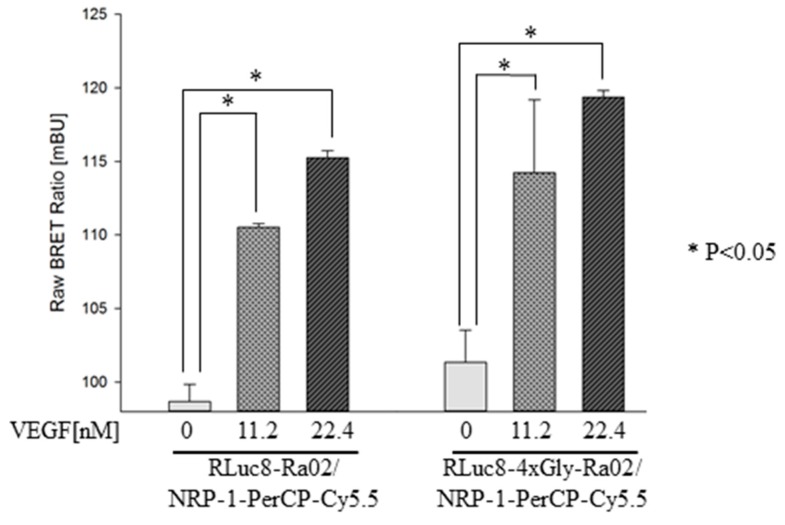
BRET ratios in dependence of the VEGF concentration. Detection of different VEGF concentrations (11.2 nM; 22.4 nM) according to Scheme 3 with simultaneous incubation. Plotted results were measured in triplicate, two unrelated biological samples were used to calculate mean values ± SD.

Finally, we had a closer look at different VEGF concentrations measured with Scheme 3, after simultaneous incubation (Figure 4). Here, again, significantly different BRET ratios compared to the no-VEGF controls were observed for both variants of donor molecules. (RLuc8-Ra02/NRP1-PerCP-Cy5.5^®^: 115.3 ± 5.0 mBU for 22.4 nM VEGF, 110.5 ± 2.0 mBU for 11.2 nM VEGF and 98.7 ± 1.2 mBU for the control; RLuc8-4xGly-Ra02/NRP1-PerCP-Cy5.5^®^: 119.4 ± 4.0 mBU for 22.4 nM VEGF, 114.2 ± 5.0 mBU for 11.2 nM VEGF and 101.4 ± 2.2 mBU for the control). 

The specificity of the BRET assay after simultaneous incubation for VEGF-A was ensured by the use of the Ra02 binding domain of the donor molecule, which is based on ranibizumab, a VEGF inhibitor used in clinics for years now to block VEGF-induced neovascularization, and the use of NRP-1, which only binds to the C-terminal part of VEGF-A. The system described here cannot distinguish between the VEGF-A splice variants containing the angiogenic terminal NRP-1 site and the VEGF receptor -1 and -2 motifs, where ranibizumab is bound [20].

In this proof-of-concept study, we added both donor and acceptor molecules in excess, but for more precise results in the future, especially for use in implantable devices, adjustments regarding donor and acceptor concentrations to this method will be necessary.

## 4. Conclusions

In summary, a two-component BRET system to detect VEGF was successfully developed. In addition, we are the first to describe that RLuc8 is able to transfer energy to the chemical fluorophore PerCP-Cy5.5^®^, which leads to a measureable BRET signal. We show that it is possible to detect VEGF in solution with simultaneous incubation of the VEGF binding donor and acceptor molecules. Our results are a small but encouraging step forward in the development of an implantable biosensor device to detect or even quantify the mitogen VEGF in vivo to monitor VEGF-induced diseases in the eye. This will be useful to adjust treatment with anti-VEGF molecules to patient needs.

## Figures and Tables

**Table 1:** Different BRET pairs used in protein research. Bioluminescence resonance energy transfer (BRET), *Renilla reniformis* luciferase (RLuc), enhanced yellow fluorescent protein (eYFP), green fluorescent protein (GFP), red fluorescent protein (RFP), fluorescent protein (FP), coelenterazine (CLZ), mutated *Renilla reniformis* luciferase (RLuc8), red-shifted *Renilla reniformis* luciferase mutant (RLuc8.6).

Name	Energy Donor	Energy Acceptor	Luciferase Substrate	Spectral Resolution ∆λem (nm)	Ref.
BRET 1	RLuc	eYFP	n-CLZ	50	[3]
BRET 2	RLuc	GFP2	CLZ400a	115	[6]
eBRET 2	RLuc8	GFP2	CLZ400a	115	[4]
BRET 3	RLuc8	mOrange	n-CLZ	85	[5]
BRET 3.1	RLuc8	mOrange	CLZ-v	50	[5]
BRET 4.1	RLuc8	TagRFP	CLZ-v	70	[5]
BRET 5	RLuc8.6	TagRFP	n-CLZ	50	[5]
BRET 6	RLuc8.6	TurboFP	n-CLZ	100	[5]
BRET 6.1	RLuc8.6	TurboFP	CLZ-v	65	[5]
**BRET 7**	**RLuc8**	**PerCP-Cy5.5**	**n-CLZ**	**300**	

## References

[1] Förster T. (1948). Zwischenmolekulare Energiewanderung und Fluoreszenz. Ann. Phys..

[2] Heyduk T., Heyduk E. (2002). Molecular beacons for detecting DNA binding proteins. Nat. Biotechnol..

[3] Xu Y., Piston D.W., Johnson C.H. (1999). A bioluminescence resonance energy transfer (BRET) system: Application to interacting circadian clock proteins. Proc. Natl. Acad. Sci. USA.

[4] De A., Loening A.M., Gambhir S.S. (2007). An improved bioluminescence resonance energy transfer strategy for imaging intracellular events in single cells and living subjects. Cancer Res..

[5] Dragulescu-Andrasi A., Chan C.T., De A., Massoud T.F., Gambhir S.S. (2011). Bioluminescence resonance energy transfer (BRET) imaging of protein-protein interactions within deep tissues of living subjects. Proc. Natl. Acad. Sci. USA.

[6] Dionne P., Mireille C., Labonte A., Carter-Allen K., Houle B., Joly E., Taylor S.C., Menard L., van Dyke K., van Dyke C., Woodfork K. (2002). BRET2: Efficient energy transfer from Renilla luciferase to GFP2 to measure protein-protein interactions and intracellular signalling events in live cells. Luminescenc Biotechnology: Instruments and Applications.

[7] Dacres H., Michie M., Wang J., Pfleger K.D., Trowell S.C. (2012). Effect of enhanced Renilla luciferase and fluorescent protein variants on the Förster distance of Bioluminescence resonance energy transfer (BRET). Biochem. Biophys. Res. Commun..

[8] Machleidt T., Woodroofe C.C., Schwinn M.K., Méndez J., Robers M.B., Zimmerman K., Wood K.V. (2015). NanoBRET A Novel BRET Platform for the Analysis of Protein-Protein Interactions. ACS Chem. Biol..

[9] Borroto-Escuela D.O., Flajolet M., Agnati L.F., Greengard P., Fuxe K. (2013). Bioluminescence Resonance Energy Transfer (BRET) Methods to study G Protein-coupled Receptor-Receptor Tyrosine Kinase Heteroreceptor Complexes. Methods Cell Biol..

[10] Wimmer T., Lorenz B., Stieger K. (2015). Functional characterization of AAV-expressed recombinant anti-VEGF single-chain variable fragments in vitro. J. Ocul. Pharmacol. Ther..

[11] Wimmer T., Lorenz B., Stieger K. (2016). Quantification of the vascular endothelial growth factor with a bioluminescence resonance energy transfer (BRET) based single molecule biosensor. Biosens. Bioelectron..

[12] Ferrara N. (2004). Vascular endothelial growth factor: Basic science and clinical progress. Endocr. Rev..

[13] Raimondi C., Fantin A., Lampropoulou A., Denti L., Chikh A., Ruhrberg C. (2014). Imatinib inhibits VEGF-independent angiogenesis by targeting neuropilin 1-dependent ABL1 activation in endothelial cells. J. Exp. Med..

[14] Muether P.S., Hermann M.M., Viebahn U., Kirchhof B., Fauser S. (2012). Vascular endothelial growth factor in patients with exudative age-related macular degeneration treated with ranibizumab. Ophthalmology.

[15] Li J., Sun K., Chen Z., Shi J., Zhou D., Xie G. (2017). A fluorescence biosensor for VEGF detection based on DNA assembly structure switching and isothermal amplification. Biosens. Bioelectron..

[16] Lin X., Leung K.H., Lin L., Lin L., Lin S., Leung C.H., Lin J.M. (2016). Determination of cell metabolite VEGF 165 and dynamic analysis of protein-DNA interactions by combination of microfluidic technique and luminescent switch-on probe. Biosens. Bioelectron..

[17] Muller Y.A., Chen Y., Christinger H.W., Li B., Cunningham B.C., Lowman H.B., de Vos A.M. (1998). VEGF and the Fab fragment of a humanized neutralizing antibody: Crystal structure of the complex at 2.4 Å resolution and mutational analysis of the interface. Structure.

[18] Geretti E., Shimizu A., Kurschat P., Klagsbrun M. (2007). Site-directed mutagenesis in the B-neuropilin-2 domain selectively enhances its affinity to VEGF165, but not to semaphorin 3F. J. Biol. Chem..

[19] Fuh G., Wu P., Liang W.C., Ultsch M., Lee C.V., Moffat B., Wiesmann C. (2006). Structure-function studies of two synthetic anti-vascular endothelial growth factor Fabs and comparison with the Avastin™ Fab. J. Biol. Chem..

[20] Dadgostar H., Waheed N. (2008). The evolving role of vascular endothelial growth factor inhibitors in the treatment of neovascular age-related macular degeneration. Eye.

